# In vitro testing of a novel implant for angular stabilisation of the posterior pelvic ring

**DOI:** 10.1186/s13018-025-06163-7

**Published:** 2025-08-07

**Authors:** Laura Zengerle, Ivan Marintschev, Christian Liebsch, Oliver Toschka, Gunther Hofmann, Hans-Joachim Wilke

**Affiliations:** 1 Institute of Orthopaedic Research and Biomechanics, Centre for Trauma Research Ulm, Ulm University Medical Centre, Helmholtzstr. 14, 89081 Ulm, Germany; 2 Katholisches Krankenhaus “St. Johann Nepomuk“ Erfurt, Erfurt, Germany; 3 Department of Trauma, Hand and Reconstructive Surgery, University Hospital Jena, Friedrich Schiller University, Jena, Germany

**Keywords:** Pelvis, Osteoporotic fractures, Insufficiency fractures, Pelvic fusion, In vitro study

## Abstract

**Background:**

Dorsal pelvic ring fractures occur in younger patients due to high-energy trauma or in elderly patients due to osteoporosis as fragility fractures of the pelvis (FFP). To date, there is no consensus on the optimal stabilization technique to treat fragility fractures of the posterior pelvic ring. The purpose of this biomechanical in vitro study was to evaluate a novel angle-stable nail system with respect to its stabilising ability in comparison with established procedures.

**Materials and methods:**

Eight fresh frozen human pelvic specimens (51–90 years, BMD 40–111 mg CaHA/cm³), were destabilised using a standardised FFP IIb fracture model according to Rommens and Hofmann (equivalent to AO/OTA 61C1.3) and consecutively stabilised with the different implant systems. The intact, fractured, and surgically treated pelvises were tested in a custom-developed pelvic test set up integrated into a universal spine tester. For simulating daily-life loading scenarios, physiological loads were applied to the specimen via the femora and four muscle pulls. The test protocol consisted of a two-legged stance in two loading conditions, respectively, which was followed by an alternating, gait-like stance testing protocol. Instability was assessed via the relative displacements at the fracture gaps.

**Results:**

The relative displacements of the fractured pelvic specimens showed significant destabilization of the pelvis compared to the intact state (*p* < 0.05) which could be reversed with the novel implant as well as with all conventional treatment techniques to a certain extent.

**Conclusions:**

Surgical instrumentation of dorsal pelvic ring fractures appear to be primary stable enough to allow immediate weight bearing. From a biomechanical point of view, the novel nail implant seems to be comparable or even slightly superior to already established procedures for stabilization of pelvic insufficiency fractures. However, the current clinical standard of treating FFP type IIb fractures with S1 screws has been biomechanically confirmed. Lumbopelvic suspension and S2 screws should not primarily be used for stabilization of dorsal pelvic fractures of type IIb or higher but may alternatively be used in patients in whom implantation of the novel implant or S1 screws is not possible due to anatomic conditions.

## Introduction

The number and frequency of pelvic fractures, also referred to as insufficiency or fragility fractures of the pelvis (FFP), is steadily increasing due to the demographic change and an ageing population [2, 13, 17]. These injuries are typically associated with prolonged hospital stays, high morbidity and mortality, and loss of independence [15, 20].

In contrast to high-energy trauma, which typically affects younger patients, pelvic fragility fractures show different patterns in elderly patients due to their lower and often osteoporotic bone quality [1]. Therefore, Rommens and Hofmann [17] proposed a special classification system for fragility fractures of the pelvis (FFP). This classification takes into account that the individual regions of the pelvis are affected at different rates: In 18% of cases (43 of 245 patients), an isolated fracture of the anterior pubic ramus was diagnosed and classified as FFP type Ia, whereas in 24% of cases (59 of 245 patients), the posterior ring was involved as well. This combination of pubic ramus fracture and non-displaced sacral fracture was classified as FFP type IIb. To relieve pain, regain mobility, and preserve the patients’ quality of life, fractures involving the posterior pelvic ring must be stabilised [11, 13, 23], because the results of non-operative treatment have not been satisfying [12].

S1 and S2 screws are commonly used for the stabilisation of the posterior pelvic ring though being associated with fixation failure and loss of reduction [5]; Salari, Moed, and Bledsoe [19]), since angular stability is not provided within the implant itself [29]. Lumbopelvic stabilisation with an internal fixator can be difficult in osteoporotic patients and is often associated with complications such as loosening or back-out of the implants, followed by loss of reduction or non-union of the fracture as well as wound complications and infections (Yilmaz et al. 2020; Jones et al. 2012; Bellabarba et al. 2006). Marintschev and Hofmann therefore developed a surgical technique using a novel implant (SACRONAIL^®^, SIGNUS Medizintechnik GmbH, Alzenau, Germany) aiming for fixed-angle stabilisation of posterior pelvic ring fractures using a minimal-invasive approach [11]. Its clinical proof of concept reported general improvement of the functional outcome with early mobilization, while long-term and experimental results are missing so far [6, 21, 26].

To date, there is no consensus on the optimal stabilization technique to treat fragility fractures of the posterior pelvic ring [8]. The current study focused on the biomechanical evaluation of this novel implant and technique [11] by using a novel biomechanical in vitro test set up and physiological test method for lumbopelvic specimens. The aim of this study was to determine the ability to stabilise posterior pelvic ring fractures using the novel implant and surgical technique and to compare it with already established surgical procedures using S1 and S2 screws and lumbopelvic screw-rod stabilization.

## Methods

### Specimens

Eight fresh frozen human cadaveric pelvis specimens with lumbosacral spine (L4-S1) and femora were obtained from donors (4 female, 4 male) with a median age of 76 years (51–90 years) and a median BMD of 87 mg CaHA/cm³ (45–132 mg CaHA/cm³) (Table 1). The use of these specimens was approved by the ethical committee board of the University of Ulm (vote no. 142/17). The sample size of *n* = 8 was based on recommendations for in vitro testing of spinal implants [25]. Quantitative computer tomography (QCT) was performed to measure the trabecular BMD. Computer tomography (CT) scans were taken to exclude fractures, tumours, and other disorders that might influence the biomechanical properties. After careful selection of the specimens, all soft tissue was removed preserving all bony structures, intervertebral discs, ligaments, and joint capsules. Screws were inserted into the cranial endplate of the upper vertebra and the caudal end of the femora before embedding in polymethylmethacrylate (PMMA, Technovit 3040, Heraeus Kulzer, Wehrheim, Germany). Flanges were mounted on the cranial PMMA embeddings for proper fixation in the testing machines. Before and after testing, specimens were stored in triple sealed polyethylene bags at -20 °C. Prior to testing, the specimens were carefully thawed overnight at +4 °C. During testing, the specimens were kept moist using 0.9% saline solution [25].

**Table 1:** Detailed information about tested specimens and implants used for the investigation of different surgical treatments for the stabilization of fractured pelvis

					Length in mm		
Specimen #	Sex	Age in years	Trabecular BMD in mg CaHA/cm³	Diagnosis on bone quality	S1 screw	Nail	S2 screw
1	f	84	90.5	osteopenia	150	145	140
2	m	64	132.1	none	170	157	140
3	f	66	40.0	osteoporosis	170	n/a	155
4	m	74	111.0	none	170	174	170
5	f	90	61.8	osteoporosis	170	151	155
6	m	89	98.2	osteopenia	160	150	140
7	m	77	83.1	osteopenia	170	170	140
8	f	51	44.6	osteoporosis	170	166	140
Median (range)		75.5 (51–90)	86.6 (44.6–132.1)		170 (150–170)	157 (145–174)	140 (140–170)

### Biomechanical testing

**Fig. 1:**
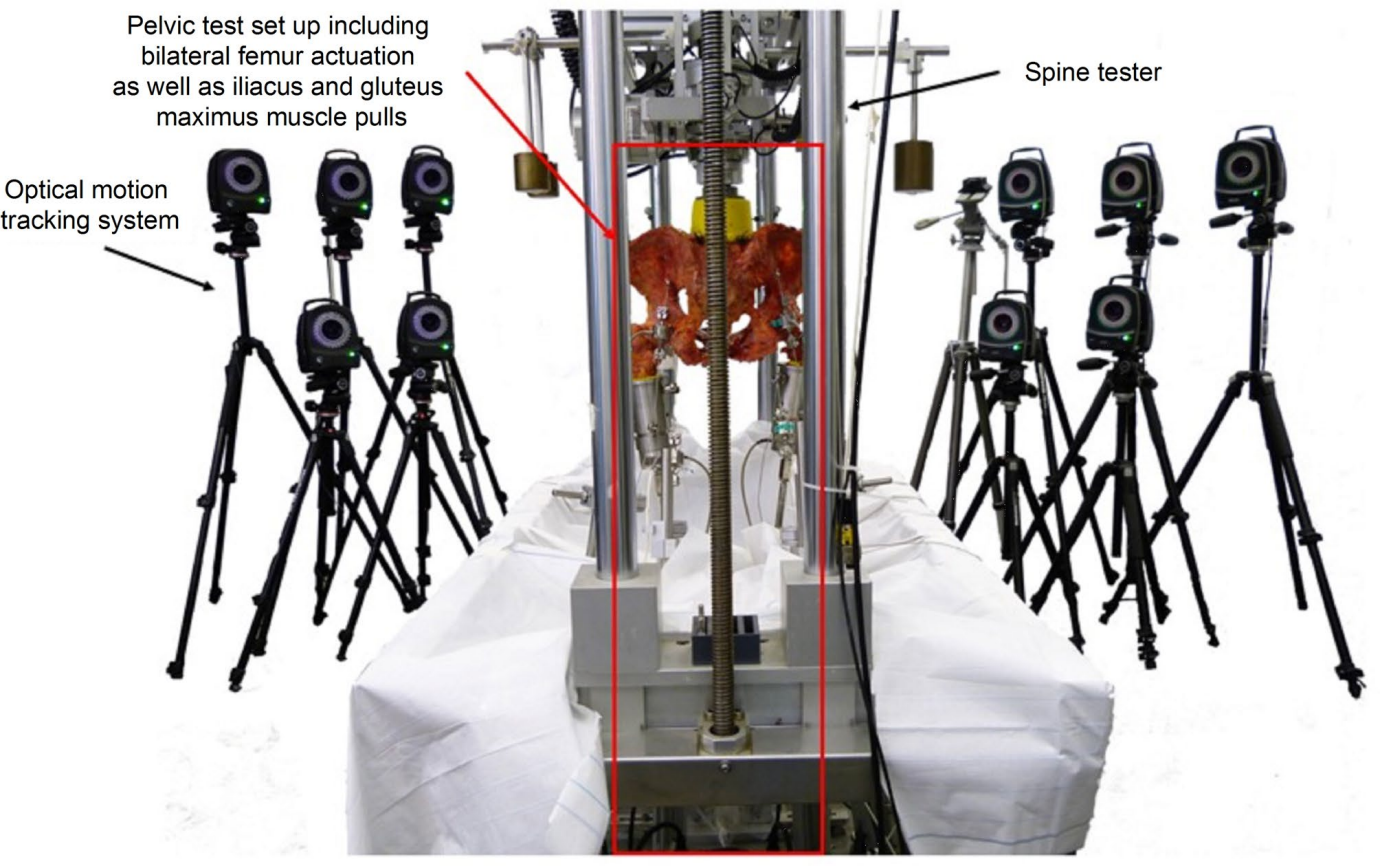
Universal spine tester [24] with the specially developed pelvis test set-up for the biomechanical evaluation of a novel nail implant for the treatment of insufficiency fractures of the pelvis

The specimens were loaded in a universal spine tester [24] and a custom-developed pelvic test set up (Fig. 1) under near-physiological conditions by applying loads to the specimen via the femora and four muscle pulls (bilateral iliacus and gluteus maximus muscles). The test protocol consisted of a two-legged stance in a lower (200 N) and a higher (300 N) loading condition which was then followed by an alternating, gait-like stance testing protocol. The two-legged stances have been performed for five loading cycles before running ten gait-like cycles. For the biomechanical evaluation of the pelvis, motion was captured using three-dimensional optical motion tracking (Vicon Motion Systems Ltd., Oxford, UK) defining one solid body by three retro-reflective markers. Relative displacements (translations and rotations) within all spinal segments, pelvic joints, and fracture gaps (interfragmentary displacements) were determined to evaluate the implants’ effects on the primary stability of the specimens. Instability was assessed by means of the relative displacements in the third full loading cycle of each loading condition.

### Fracture model and surgical treatments

**Fig. 2:**
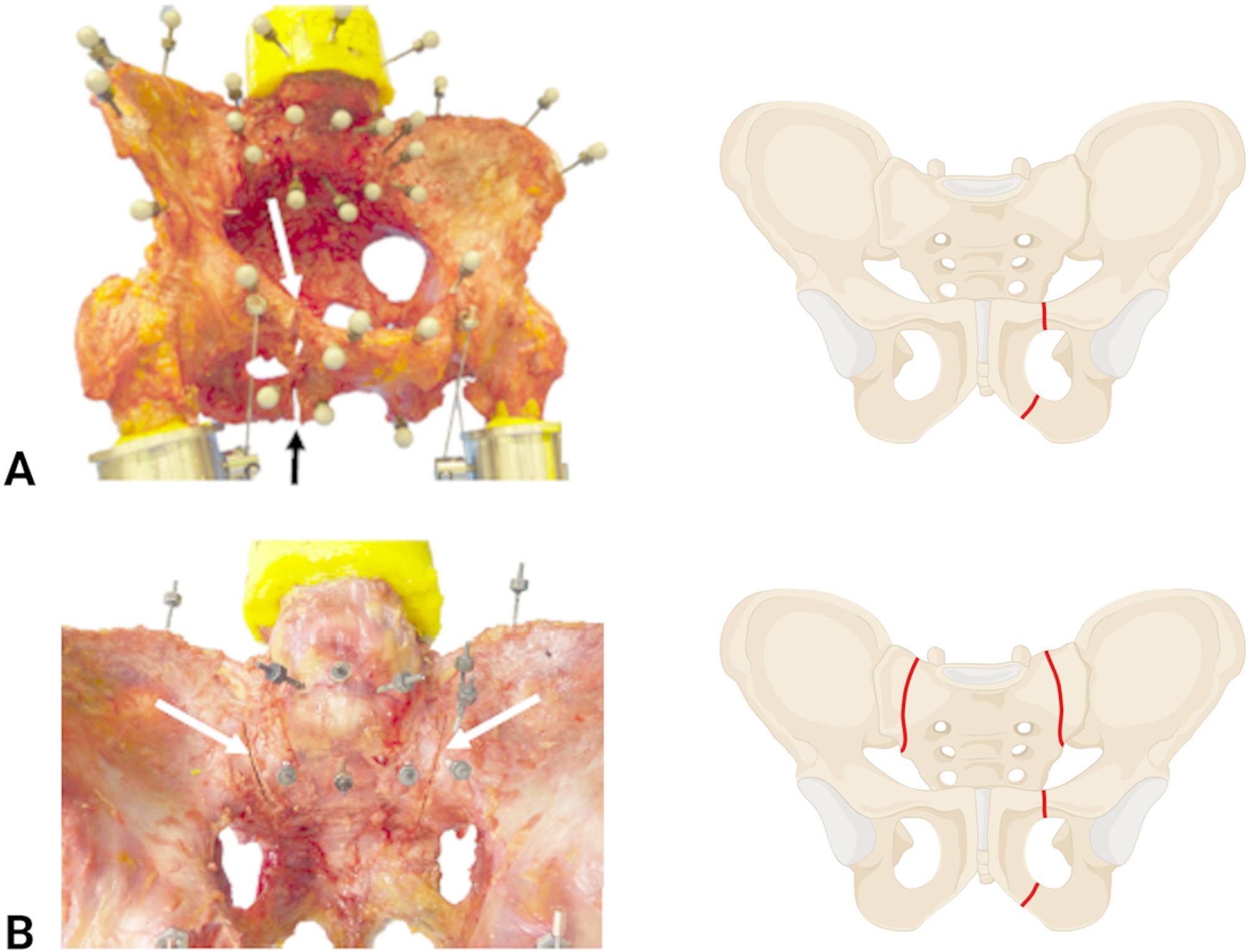
(**A**) FFP type Ia model consisting of a fracture of the pubic bone (fracture gaps marked with arrows in white and black) according to Rommens and Hofmann [17]. (**B**) FFP type IIb model consisting of a fracture of the pubic bone and a bilateral alar fracture (fracture gaps marked with arrows in white), according to Rommens and Hofmann. Created in BioRender. Greiner-Perth, A. (2025) https://BioRender.com/ g28g652

After assessing the intact biomechanical properties of the specimens, a standardised fracture was artificially created in the pelvis using an oscillating saw. First, only the anterior pelvic ring was cut in the pubic bone, which is classified as fragility fracture of the pelvis (FFP) type Ia according to Rommens and Hofmann [17] (Fig. 2a) and the range of motion and relative displacement of the isolated fracture were determined. Subsequently, a bilateral alar fracture was set with an oscillating saw which resulted in a FFP type IIb (equivalent to AO/OTA 61C1.3) (Fig. 2b). The biomechanical properties could not be evaluated for the FFP type IIb model due to resulting extensive instability found during preliminary testing.

### Surgical treatments

**Fig. 3:**
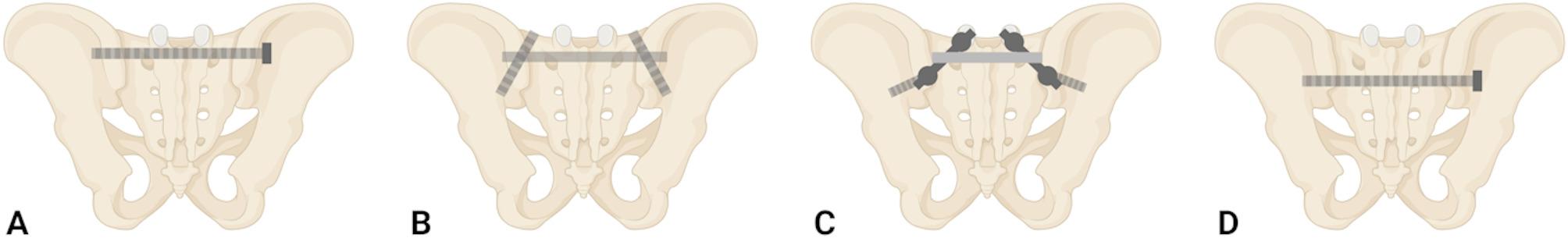
Schematic view of the four surgical treatment options in the order of implantation: (**A**) S1 screw, (**B**) nail, (**C**) lumbopelvic fixation, (**D**) S2 screw. Created in BioRender. Greiner-Perth, A. (2025) https://BioRender.com/ d86u868

The different systems that are clinically used for the surgical treatment of insufficiency fractures of the pelvis were consecutively implanted into the specimens after setting the standardized FFP type IIb fracture (Fig. 3). The correct sizes of the implants were preliminary chosen based on CT scans for each individual specimen. The implantation of all systems was performed by two advanced trauma surgeons specialized in pelvic surgery using system-specific instruments. Screw placement with regard to insertion angles and trajectory heights was performed in accordance with the operating surgeons’ extensive clinical experience and current best clinical practice. Prior to each next testing step, the implants were carefully removed.

**Fig. 4:**
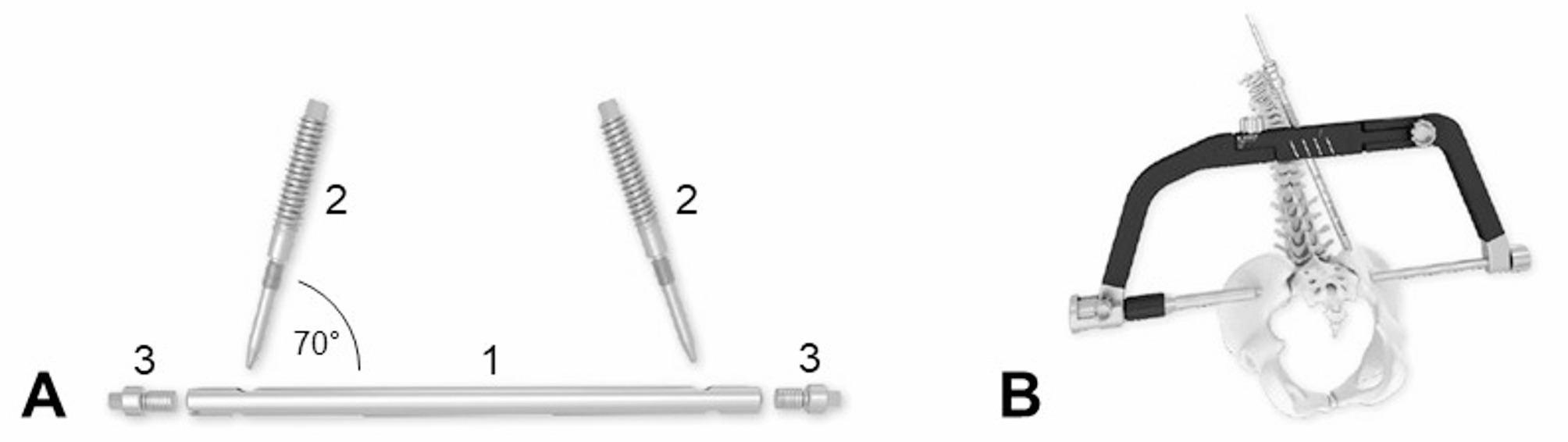
(**A**) Illustration of the novel nail system SACRONAIL^®^ (1 = Nail, 2 = Locking screws, 3 = Optional screw plugs). (**B**) Illustration of the guide bracket with mounted instrument guide for correct locking screw placement. Kindly provided by SIGNUS Medizintechnik GmbH

First, a cannulated screw (*S1 screw*, DePuy Synthes, Zuchwil, Switzerland) with an outer diameter of 7.3 mm and a long thread of 32 mm length was implanted through the corridor of S1 (Fig. 3a). The total length of the screw was adapted to the individual specimen (Table 1). The nail of the novel *nail* system (SACRONAIL^®^, SIGNUS Medizintechnik GmbH, Alzenau, Germany) with a diameter of 8 mm was placed in the same corridor of S1 (Fig. 3b). The novel implant system consists of one nail and two locking screws made of Ti-6Al-4 V with a 70-degree angle each and was developed to provide high rotational stability (Fig. 4a). The angular-stable connection between locking screws and nail is achieved through a form- and force-locked fixation via a combination of machine-threaded screws and a cylindrical peg-in-bore interface. The nail length was chosen with respect to the individual specimen, while lengths between 135 and 149 mm were available; as this parameter was considered important during the experiments at first, the length value of one nail implant was not documented (Table 1). Two ilium screws with a diameter of 7.5 mm, a ventral length of 30 mm, and a dorsal length of 60 mm were then bilaterally implanted into the iliac crests using a guide bracket with mounted instrument guide (Fig. 4b). In the next step, the *lumbopelvic fixation* system (DIPLOMAT, SIGNUS Medizintechnik GmbH, Alzenau, Germany) was instrumented from L5 to Ilium (Fig. 3c). For this, two pedicle screws (length x diameter: 45 mm x 6.5 mm) were cross-connected with two ilium screws (length x diameter: 100 mm x 9.5 mm) by a rigid rod. In the last step, another cannulated screw with the corridor-specific length was implanted into the corridor of S2 (*S2 screw*) (Fig. 3d). Finally, the specimens were tested after removal of all material.

### Statistical analysis

Statistical analysis was performed using SPSS (IBM^®^ SPSS^®^ Statistics Version 24; IBM Corp., Armonk, NY) for the displacement values in all motion planes in every testing condition. Normal distribution could not be proven by Shapiro-Wilk test with a significance level of 0.05. Therefore, median and ranges are provided for all data and non-parametric tests were performed. Significant differences were assessed using the Friedman test with Bonferroni post-hoc correction and a significance level of α = 0.05.

## Results

**Fig. 5:**
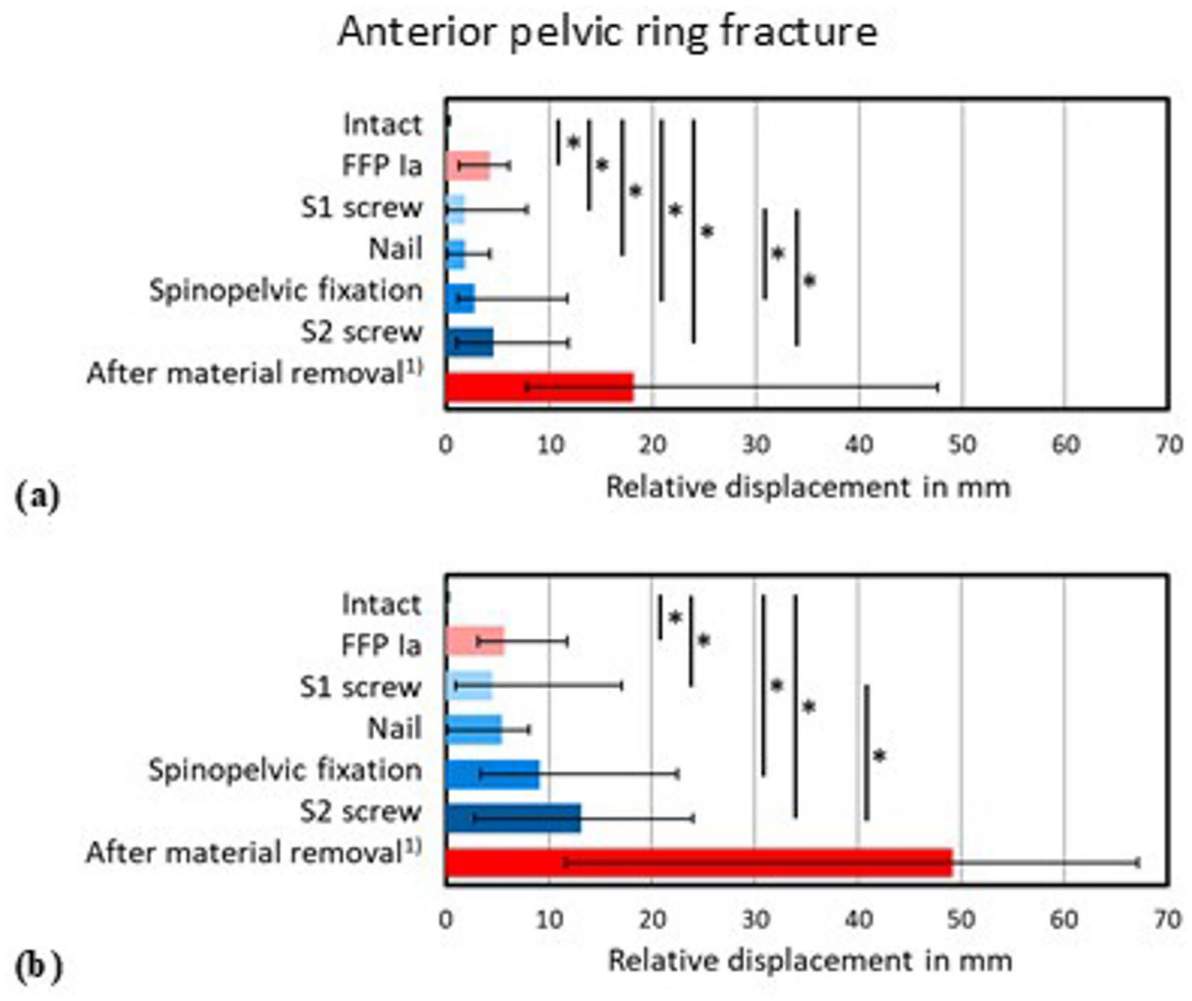
Relative displacement in mm of the fracture gap of the anterior pelvic ring fracture during simulation of the (**a**) lower and (**b**) higher loaded stance in the intact and fractured condition (FFP Ia), after implantation of the S1 screw, nail, lumbopelvic fixation and S2 screw into a FFP IIb fracture, and after material removal, 1) with a loss of 3 specimens, so that no statistical analysis could be performed for this condition. * *p* < 0.05

The FFP Ia model led to a significant (*p* < 0.05) destabilization of the pelvis due to large displacements in the fracture gap of the anterior pelvic ring lesion during the simulation of the stance phase (Fig. 5). With the implantation of the different implants in the FFP type IIb fracture model, primary stability of the anterior pelvic ring fracture condition could be generally restored. In the lower loading case (200 N), all implants showed a significant (*p* < 0.05) difference towards the intact condition (Fig. 5a). Furthermore, the difference between the S1 screw and the lumbopelvic fixation or the S2 screw, respectively, was also statistically significant. After implantation of the nail system with a median relative displacement of 5.43 mm in the higher loading case (300 N), there was even no significant difference (*p* > 0.05) compared to the intact condition, while all other implants showed a significant difference (*p* < 0.05) compared to the intact pelvis (Fig. 5b). However, the median relative displacements for the S1 screw decreased remarkably to only 4.55 mm. After performing the lumbopelvic fixation, median relative displacement increased to 9.11 mm and after implantation of the S2 screw to even 13.12 mm, which both was significantly (*p* < 0.05) larger compared to the treatment using the S1.

**Fig. 6:**
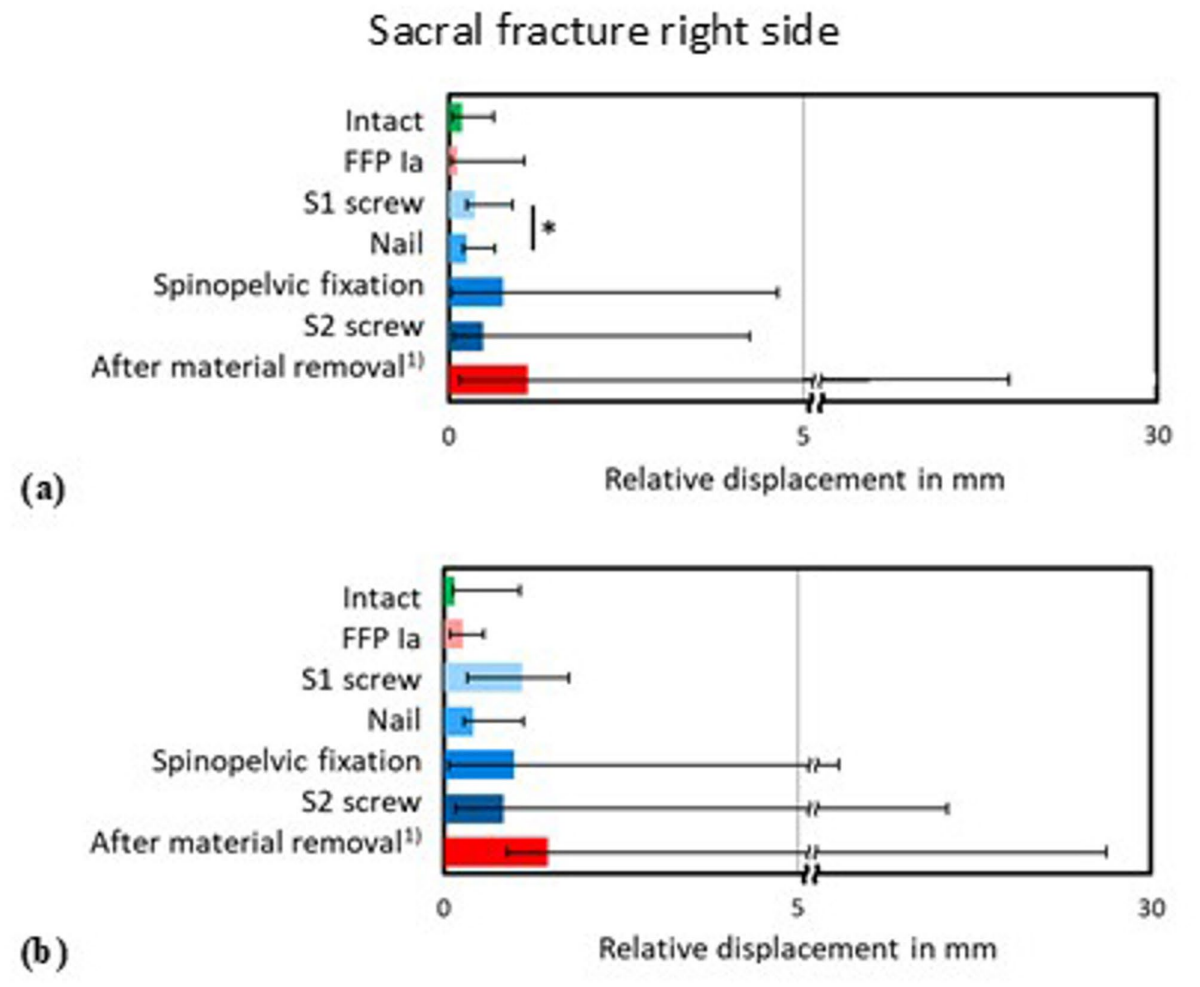
Relative displacement in mm of the fracture gap of the right sacral fracture during simulation of the (**a**) lower and (**b**) higher loaded alternating stance in the intact and fractured condition (FFP Ia), after implantation of the S1 screw, nail, lumbopelvic fixation and S2 screw into a FFP IIb fracture and after material removal, 1) with a loss of 3 specimens, so that no statistical analysis could be performed for this condition. * *p* < 0.05

**Fig. 7:**
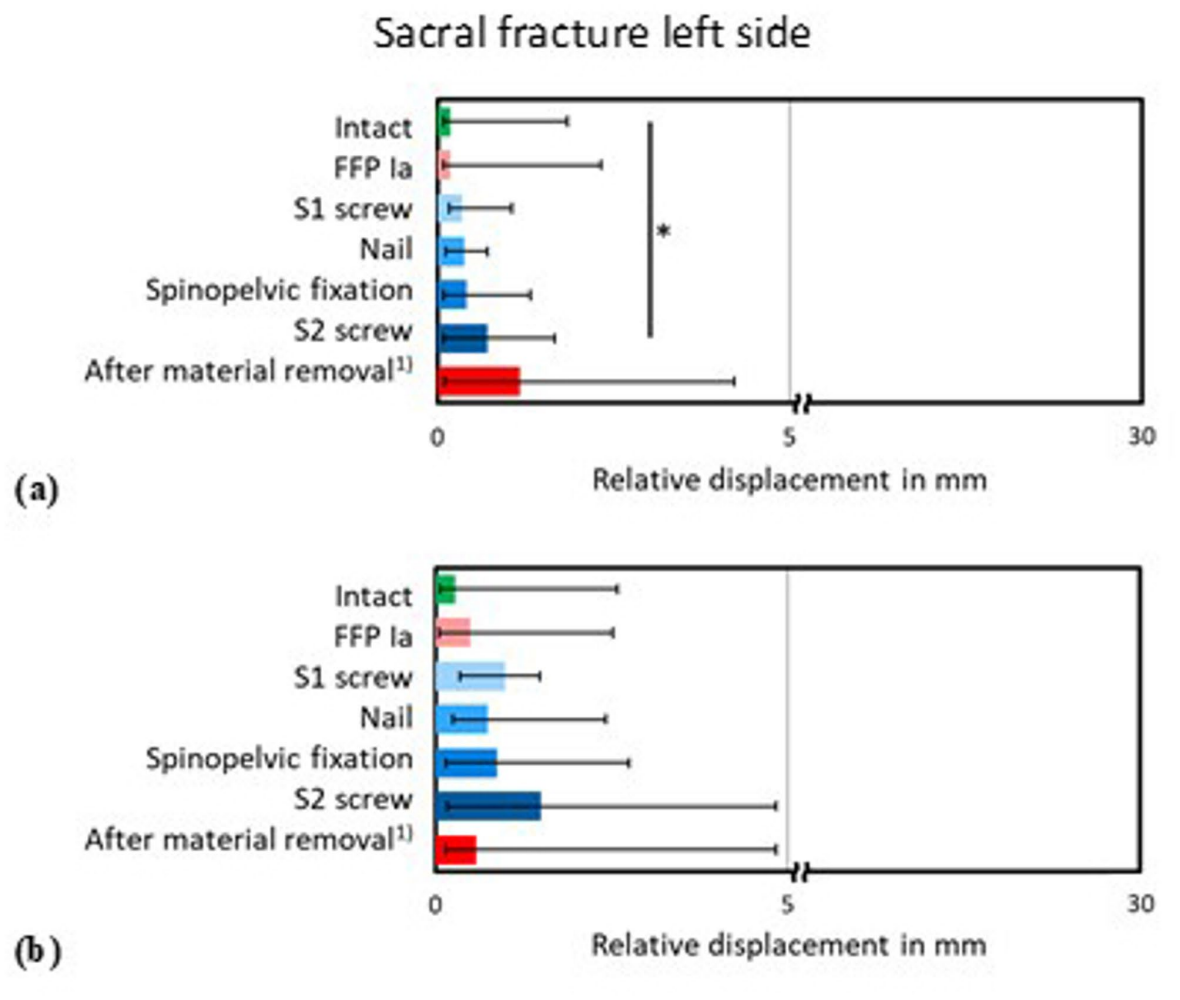
Relative displacement in mm of the fracture gap of the left sacral fracture during simulation of the (**a**) lower and (**b**) higher loaded alternating stance in the intact and fractured condition (FFP Ia), after implantation of the S1 screw, nail, lumbopelvic fixation and S2 screw into a FFP IIb fracture and after material removal, ^1)^ with a loss of 3 specimens, so that no statistical analysis could be performed for this condition. * *p* < 0.05

Displacements of the bilateral sacral alar fractures were evaluated during the simulation of the gait-like alternating stance (Figs. 6 and 7). For the lower loading condition (200 N), the fracture stabilisation with the novel nail implant system (median: 0.25 mm, range: 0.44 mm) was significantly (*p* < 0.05) higher than with the S1 screw (median: 0.37 mm, range: 0.65 mm) at the right side of the pelvis (Fig. 6a). For the lumbopelvic fixation and the S2 screw, the destabilisation increased again, whereas the S2 screw led to a smaller destabilisation compared to lumbopelvic fixation. Simulation of the alternating stance with higher loading conditions (300 N) showed similar relationships for all treatments with overall slightly increased relative displacements which did not result in significant changes (*p* > 0.05) between any of the tested conditions (Fig. 6b).

A comparable trend of stabilization and destabilization of the fracture gaps could be evaluated in the alar fracture at the left side in the alternating stance phases with both the lower (200 N, Fig. 7a) and the higher (300 N, Fig. 7b) loading condition, respectively. Significant (*p* < 0.05) destabilization compared to the intact condition was solely found for the S2 screw after implantation in the FFP type IIb simulating the lower loaded alternating stance.

## Discussion

The aim of this study was to biomechanically evaluate a novel nail implant system in comparison with three other fixation techniques that are broadly used in the clinical routine for the stabilisation of fragility fractures of the pelvis: S1 screws, S2 screws, and lumbopelvic fixation. The results of the relative displacements in the fracture gaps indicate that, from a pure biomechanical point of view under near-physiological loading conditions and testing scenarios, the novel nail implant and the S1 screw have the highest ability for the stabilisation of type IIb fragility fractures. The novel nail system even appeared to be slightly superior to the S1 screw.

In the clinical routine, the interfragmentary movement is highly relevant for fracture healing, as interfragmentary movements of more than 5 mm delay or even prevent secondary bone healing [16, 28]. Conversely, a pubic ramus fracture with a relative interfragmentary movement of less than 5 mm could potentially heal without additional stabilisation, provided that the pain is tolerable for the patient [27]. In the experimental setup of this study, the relative displacements represent this parameter of interfragmentary movement. In the lower loading scenario, the interfragmentary movement could be limited to the threshold of 5 mm after stabilisation of the fracture irrespective of the treatment. Since only the S1 screw and the novel nail showed no significant change in the direct implant comparison, it can be concluded that the difference between these two implants can be classified as small.

In the higher loading scenario, an intrafragmentary movement below the threshold was only observed after implantation of the S1 screw. However, with the novel nail implant system, this limit was only slightly exceeded while showing a lower range of the relative displacements over all specimens and there was no significant difference compared to the intact condition anymore. Based on these biomechanical findings, it can be suggested that early mobilisation, a key treatment goal to maintain the patients’ functional independence [7, 10], is possible in patients where an FFP type IIb is stabilized with the novel nail system. However, the feasibility and optimal timing of early mobilization have to be determined in clinical studies. Furthermore, after stabilisation of the fracture with the novel nail system as well as with the S1 screw, the interfragmentary movement of the pubic fracture could be limited to 5 mm so that additional stabilisation of the anterior pelvic ring could be avoided for these treatments in the clinical setting.

Since the upper limit of five millimetres of interfragmentary movement was clearly exceeded after lumbopelvic stabilisation and with S2 screw, these two implants do not represent an adequate stabilisation for pelvic fractures from a biomechanical point of view if the anterior pelvic ring is involved. However, lumbopelvic suspension may be the only possible fracture stabilization method if the transverse iliosacral corridor is too narrow or even not existent, impeding the implantation of S1 or S2 screws. As the S2 corridor is most often used in the presence of dysmorphisms in the lumbosacral region, the effects of S2 fixations should be further evaluated in future studies using the test setup proposed in this study.

Except for the results at the fracture gap of the left sacral fracture, the results of the relative displacements of the novel nail system show the smallest range. At the same time, the median of the relative displacements of the nail system is only slightly above the median of the S1 screw in one half of the cases and even significantly below it in the other half. From a biomechanical point of view, the small range of the measured values and the widely lower median values favour the nail implant as a stabilization method for dorsal pelvic fractures. This assessment is also supported by the statistical findings. However, the results also confirmed that the S1 screw represents an adequate technique. With regard to the high median relative displacements and large ranges found for the lumbopelvic stabilization and the S2 screw, these techniques should not be primarily used for the stabilisation of dorsal pelvic fractures.

To compare the stabilisation properties of the novel nail implant, three stabilisation methods commonly used for osteoporotic fractures were selected (Gansslen et al. [3; Rommens et al. [4, 18, 22]. The aim of each of these stabilisation methods is early mobilisation of the mostly elderly patients under full weight-bearing [9, 14]. By testing all implants subsequently in all pelvic specimens, all implants could be directly compared with each other. The sequence of the individual test steps was selected in ascending order according to the implant diameter, so that it can be assumed that any microstructural bone changes in the area around the implant were eliminated by the subsequent reaming. Potential dependency of the results on the testing order is therefore a limitation of this study. However, further destabilisation of the bony construct due to the implantation technique inhibited randomization. In addition to these limitations, several further constraints have to be acknowledged. One key limitation of this study is the exclusive focus on a single fracture model (FFP Type IIb), which limits the generalizability of the findings to more complex or unstable fracture patterns, such as FFP Types III or IV. Future studies should incorporate a broader range of fracture configurations to better reflect clinical heterogeneity and to evaluate implant performance under more challenging biomechanical conditions. Moreover, the potential role of S2 screw fixation remains insufficiently explored. In the present study, the novel implant was tested solely with anchorage in S1; however, extension into S2 may offer additional stability, particularly in cases of compromised bone quality in the sacral alae. Further biomechanical testing is required to assess whether bilateral or targeted S2 fixation confers advantages in terms of construct stability and load distribution.

In conclusion, surgical instrumentation of dorsal pelvic ring fractures exhibited adequate primary stability in this in vitro study, while the novel nail implant showed comparable or even slightly superior biomechanical properties to already established procedures for stabilization of pelvic insufficiency fractures. While the current clinical standard of treating FFP type IIb fractures with S1 screws could be biomechanically confirmed, lumbopelvic suspension and S2 screws showed reduced primary stability and should primarily be used in patients in whom implantation of the novel implant or S1 screws is not possible due to anatomic conditions. These findings demonstrate promising biomechanical properties of the tested implants within the defined model. Nevertheless, future evaluations should include additional fracture types, varying bone densities, age-related changes, and long-term cyclic loading protocols to comprehensively assess implant reliability under clinically realistic conditions.

## Data Availability

Data is provided within the manuscript or supplementary information files.
